# Integrated Fabrication of Novel Inkjet-Printed Silver Nanoparticle Sensors on Carbon Fiber Reinforced Nylon Composites

**DOI:** 10.3390/mi12101185

**Published:** 2021-09-29

**Authors:** Büşra Karaş, Vimanyu Beedasy, Zhaoyuan Leong, Nicola A. Morley, Kamran Mumtaz, Patrick J. Smith

**Affiliations:** 1 Innovative Process Laboratory, Department of Mechanical Engineering, The University of Sheffield, 64 Garden Street, Sheffield S1 4BJ, UK; bkaras1@sheffield.ac.uk (B.K.); k.mumtaz@sheffield.ac.uk (K.M.); 2 Applied Inkjet Printing Laboratory, Department of Mechanical Engineering, The University of Sheffield, 64 Garden Street, Sheffield S1 4BJ, UK; vbeedasy1@sheffield.ac.uk; 3 Department of Materials Science and Engineering, The University of Sheffield, Sheffield S1 3JD, UK; z.leong@sheffield.ac.uk (Z.L.); n.a.morley@sheffield.ac.uk (N.A.M.)

**Keywords:** inkjet printing, cross-cut test, printed electronics, strain sensor, carbon fiber reinforced polymers

## Abstract

Inkjet-printing technology enables the contactless deposition of functional materials such as conductive inks on surfaces, hence reducing contamination and the risk of substrate damage. In printed electronics, inkjet technology offers the significant advantage of controlling the volume of material deposited, and therefore the fine-tuning of the printed geometry, which is crucial for the performance of the final printed electronics. Inkjet printing of functional inks can be used to produce sensors to detect failure of mechanical structures such as carbon fiber reinforced composite (CFRC) components, instead of using attached sensors, which are subject to delamination. Here, silver nanoparticle-based strain sensors were embedded directly in an insulated carbon-fiber laminate by using inkjet printing to achieve an optimized conductive and adhesive geometry, forming a piezoresistive strain sensor. Following the inkjet-printing optimization process, the sensor conductivity and adhesion performance were evaluated. Finally, the sensor was quantified by using a bending rig which applied a pre-determined strain, with the response indicating an accurate sensitivity as the resistance increased with an increased strain. The ability to embed the sensor directly on the CFRC prevents the use of interfacial adhesives which is the main source of failure due to delamination.

## 1. Introduction

Inkjet printing is a widely used deposition technique, which was initially employed in the graphics industry [1] and became popular in manufacturing fields due to the advances in the composition of functional inks. Nowadays there are many published works describing the use of inkjet printing as a tool for deposition of a wide variety of inks ranging from polymers [2,3,4], ceramics [5,6], cellular inks [7,8], graphene [9], to metal-based inks [10,11,12,13,14,15,16,17]. Printed electronics can be manufactured via inkjet deposition technology by utilising functional inks printed on a variety of substrates, thereby reducing the production costs of electronic devices as compared to traditional expensive lithography techniques.

Once a functional conductive pattern is printed and thermal energy is input into the system to improve the conductivity, the performance is first assessed by a visual inspection to detect any surface deformation or cracks, followed by a resistivity and adhesion test to determine whether the pattern is suitable for the purpose of printed electronics. An adhesion test is often overlooked in the field of printed electronics, but it is crucial to evaluate this property for understanding the interface between the printed pattern and the substrate, especially for multi-layered patterns. This additional step of evaluating the adhesion is crucial for understanding the lifespan of the printed feature, particularly when it will be exposed to external harsh environments, which may cause defects or delamination due to excessive deformation.

One of the applications of the inkjet-printed sensors can be targeted towards the structural health monitoring of carbon fiber reinforced composites (CFRC) which consist of a network of fibers bound by polymers. CFRCs are highly sought in applications where mechanical strength is key to performance whilst minimising the weight of the structure, for example an aerospace fuselage or automotive chassis. Nowadays, manufacturers are prioritising the use of composites due to their reduction in weight without compromising the high strength and stiffness of the structure, with the aim of reducing the carbon emissions through decreased fuel consumption. The structural health monitoring (SHM) of CFRCs is crucial to predict the failure of a particular component [18], and this can be done via the combined use of non-destructive testing and sensors monitoring the parts during real-time operation. By exploiting these technologies, manufacturers can shift the timing of maintenance from a “scheduled” perspective to a “predicted” perspective by relying on the sensor data to prevent catastrophic failure, and hence provide time and cost-saving opportunities, with applications ranging in aerospace, mechanical and civil communities.

However, SHM techniques require the use of expensive equipment such as piezo-electric sensors, fiber-optic sensors [19,20] or ultrasonic sensors to name a few [21,22] and they can be difficult to integrate within complex systems due to their geometry and data acquisition cross-talks with other components [23]. An alternative is to use embedded sensors directly onto the carbon-fiber matrix with the aim of detecting structural changes within the laminates prior to failure [24,25]. Strain sensors can be integrated within the carbon-fiber manufacturing process, with the aim of improving the self-sensing efficiency as compared to attached sensors. In the case of strain sensors, they can easily be attached to the surface of the carbon fiber reinforced polymer (CFRP) which can be conducting or insulating, although the bonding to the host structure is the main source of failure. As attached sensors, also known as film sensors, are subject to degradation and delamination in high-stress environments, embedded sensors improve the fidelity of the sensor performance by minimising the use of polymer films and adhesive interfacial layers [26].

Rocha et al. [27] emphasized on the delamination of film sensors to their host structure as the most common failure point, then lack of sensing performance and signal fidelity. This can be overcome by inkjet printing the sensors directly on the structure. Hence, this study describes the embedding of an inkjet-printed silver sensor from a nanoparticle-based ink directly in a CFRP laminate to ensure the sensor measures the real-time deformation together with the CFRP instead of separate measurements arising from delamination. Prior to the embedding of the sensor on the CFRP, preliminary investigations into the insulator performance on top of the CFRP laminate was done. Then, an adhesion characterisation of the printed silver ink to the carbon fiber laminate was quantified through the ASTM F1842-15 standard for printed electronics. Following this, an optimisation of the sintering process to improve the conductivity of the silver ink was done, and compared to the bulk conductivity of silver. Finally, the sensor performance through piezoresistivity (change in resistance with strain) was analyzed and reported.

## 2. Materials and Methods

### 2.1. Composite Sample Fabrication

Long carbon fiber reinforced thermoplastic composite parts were fabricated using a Composite Based Additive Manufacturing technique [28]. Non-woven 14.175 *gsm* carbon fiber surfacing veil supplied by ACP Composites Inc. (Livermore, CA, USA) were used. The carbon fiber sheets were cut with a size of 210 mm × 148 mm (A5) and fed to a HP Deskjet Plus 4130 inkjet printer (Hewlett-Packard, Palo Alto, CA, USA) to print a 150 × 25 mm^2^ rectangular geometry. PA2200 nylon powder supplied by EOS GmbH (Krailling, Germany) were spread out on the printed area, resulting in a coating of nylon over the printed area after the dry excess powder was removed. This process was repeated until the last layer of carbon fiber was stacked. 50 layers of carbon fiber sheets coated with the polymer powder were stacked on top of each other, and a compacted laminate was formed by applying 8 KN of pressure for 60 min using a hot press. The hot press chamber was heated to 200 °C to allow the polymer powder to melt completely. After 60 min, the sample was left to cool under pressure at ambient conditions and the compressed part was removed from the hot press. Finally, a sand blasting process was used to remove the excess carbon fiber layers which were not coated with nylon powder, resulting in a CFRP part measuring 150 × 25 × 4 mm^3^ being manufactured.

### 2.2. Inkjet Printing

A silver nanoparticle-based (NPs) ink (Sigma-Aldrich, no. 736465, St. Louis, MO, USA) containing 30–35 wt.% of silver NPs was printed to form conductive patterns in the form of a sensor after sintering. 

An insulator ink was printed to prevent the formation of interconnects between the conductive carbon fiber laminates and the printed sensor. The insulator ink was purchased from a commercial supplier (DM-INI-7003, Dycotec Materials Ltd., Calne, UK) and was specially designed for inkjet printing applications as well as providing a smooth, hydrophilic surface for overprints using other types of inks, hence eliminating the requirement for additional surface tailoring prior to printing the silver layer. The insulator ink consists of a multifunctional diacrylate crosslinker and an oligomer to promote the UV curing process.

**Figure 1:**
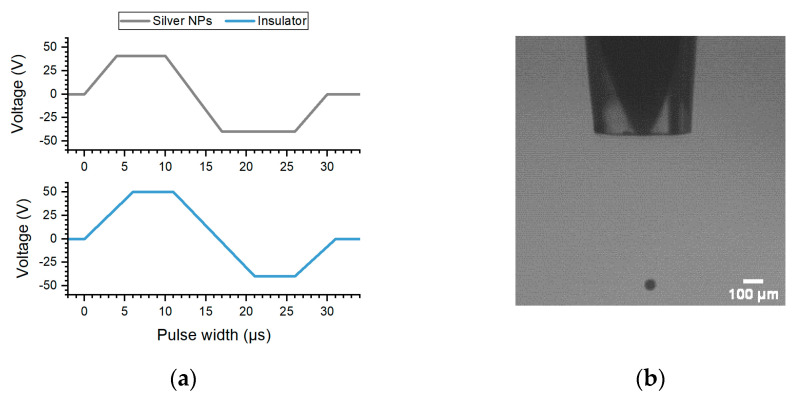
(**a**) Illustration of the waveform used to print both inks using a 60 μm printhead heated at 40 °C; (**b**) a snapshot of the droplet measuring an in-flight diameter between 40–45 μm.

The viscosity of both inks as a variation of temperature is shown in Appendix A. The insulator ink has a much higher viscosity at room temperature (25 °C) as compared to the silver ink, and hence inkjet printing was done using a heated printhead at 40 °C. At this temperature, the viscosity of the silver ink measured 11.25 mPa·s and the insulator ink 16.0 mPa·s. Hence, the printing waveform was further optimised and shown in Figure 1a below, resulting in a droplet measuring an in-flight diameter of 40–45 µm as shown in Figure 1b. 

**Figure 2:**
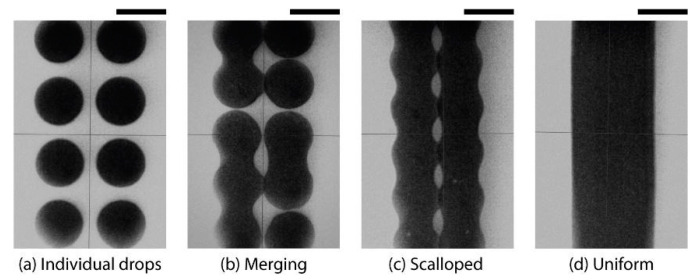
Snapshot of the droplet spacing optimisation. (**a**) represents individual drops; (**b**) randomly merged drops; (**c**) scalloped track; and (**d**) uniform track. The droplet spacing decreases from left to right. Scale bar is 50 μm.

A drop-on-demand inkjet printer (Jetlab-4XL, from MicroFab Technologies Inc., Plano, TX, USA) with a 60 µm nozzle was used. The insulator layer was printed with a droplet spacing of 30 µm, while the silver nanoparticle layer was printed on the insulator with a droplet spacing of 80 µm with two layers to ensure an even smooth coverage and optimum print resolution, while reducing the possibility of shorts within the sensor. The printing bed was heated to 50 °C to create a pinned edge after deposition. Figure 2 represents the droplet spacing optimisation process using silver NPs ink to yield a uniform track with an optimum resolution. As the droplet spacing is decreased from Figure 2a–d, the droplets on the pattern merge to form a uniform smooth track with well-defined edges.

**Figure 3:**
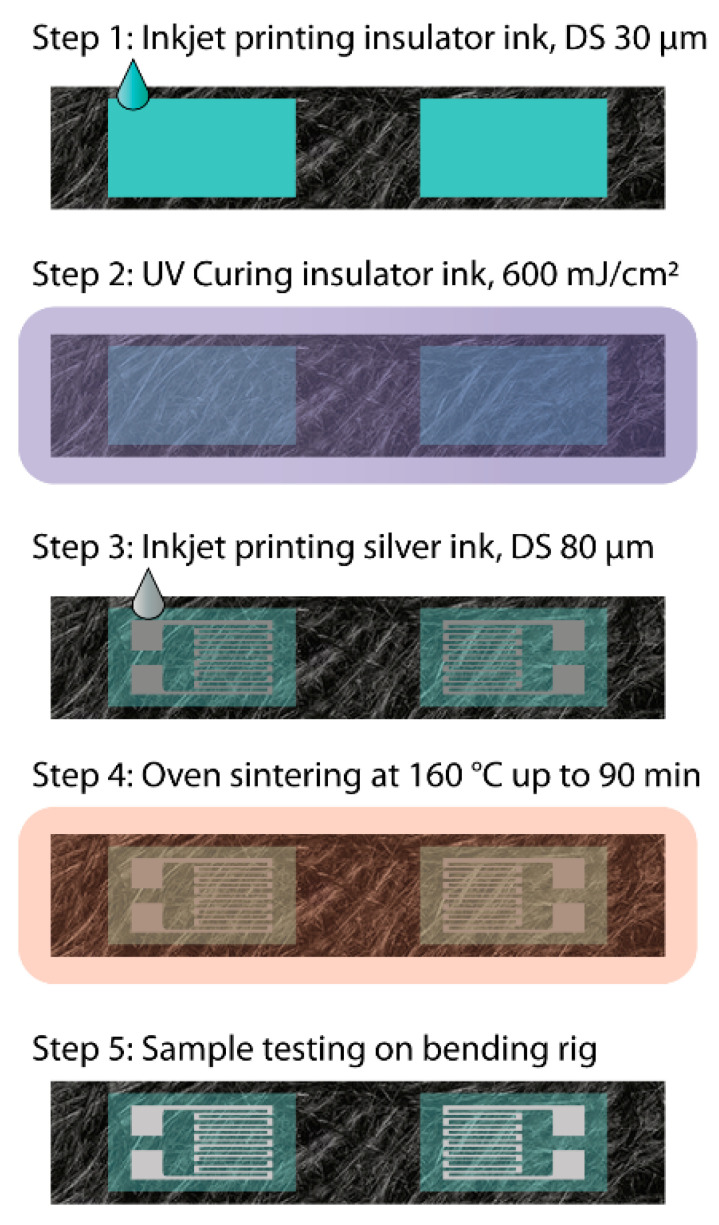
Illustration of the process steps using inkjet printing to integrate the strain sensor into the carbon fibre composite.

Once the droplet spacing was optimized for both the insulator and the silver ink, the fabrication process was carried out as per the illustration shown in Figure 3 below.

### 2.3. UV Curing 

The printed insulator ink contains a liquid oligomer with a photoinitiator, which requires UV energy to cross-link into a stable insulative layer. The curing energy recommended by the supplier, ranges between 500–1000 mJ/cm^2^ and requires incident radiation between 380–390 nm for optimum curing. The energy was supplied using a UV LED curing lamp from Phoseon Technology Inc. (Hillsboro, OR, USA) with a peak at 395 nm. The irradiance of the UV lamp at the peak wavelength can reach a maximum of 4 W/cm^2^ when the target material is placed within 10 mm of the output window of the lamp. The power output of the lamp was set to 25%, which corresponds to an output irradiance of 600 mJ/cm^2^. The duration of the applied UV energy was set to 15 s to ensure the printed insulative layer had absorbed enough energy to cross-link.

### 2.4. Silver Nanoparticle Sintering

The sintering time of the silver ink was varied between 5–90 min, with the aim of finding the optimum sintering time to maximise the sensor performance. The temperature of sintering was determined by the melting point of the PA2200 nylon powder, which was 172 °C in this case. 

### 2.5. Conductivity Evaluation Using a Four-Point Probe

To evaluate the performance of the samples two tests were performed on square test samples measuring 15 × 15 mm^2^. The first test measured the conductivity of the samples using the four-point probe technique to eliminate any lead or contact resistance between the probes and the sample. The loading of the probes onto the sample was done using a custom-built motorised Z-stage to avoid “scratch” damage to the samples and to ensure an even pressure is applied. A schematic of the rig is shown in Appendix B.

### 2.6. Adhesion Evaluation According to the F1842-15 Standard

The second test was to evaluate the adhesion of the samples according to the ASTM F1842-15 standard using scotch tape. It is a standardised test method for determining the ink adhesion on substrates for printed electronic devices, and it is based on the ASTM D3359 standard [29]. In this method, a multi-blade cutter with a cutting-edge angle of 20.6 ± 0.1°, made six cuts at once through the sample, separated by 2 mm. The sample is then brushed off for any loose particles from the test area, and a second identical cut is placed perpendicular to the first in order to achieve a grid pattern. The tape is then firmly applied to the cut sample, ensuring good contact is made with the specimen, and it is removed 90 s after application by steadily peeling it off back upon itself as close to a 180° angle as possible. A schematic of the cutting tool and the procedure is provided in Appendix C.

The inspection of the adhesion results was done through digital image processing to avoid the human assessment bias, as explained by Lukacs et al. [30]. The samples are first photographed using a high-resolution camera, followed by a monochrome conversion and the areas of delamination quantified as “voids” from the overall selected sample area. This methodology provided an unbiased and more reliable assessment of the adhesion. The delaminated area was then compared to the standard test results shown in the ASTM F1842-15 standard [31], which is reproduced in Appendix D.

### 2.7. Thickness of Inkjet-Printed Samples

The thickness of the printed silver samples was also evaluated using atomic force microscopy (Nanoscope IV Multimode AFM from Kroto Research Institute, Sheffield, UK) in the tapping mode, with the aim of quantifying the resistivity of the printed ink for comparison with the bulk resistivity of silver.

### 2.8. Sensor Performance Characterization

The resistance changes in the coils were measured using a BK879B LCR meter (BK Precision, Yorba Linda, CA, USA) in the resistance mode; the data was collated on a PC through its serial output, and 4 measurements were recorded per second. Prior to each application of stain, the initial sensor resistance was measured, followed by the application of strain for 10 s using the bending rig of test radii 300, 400, 500 and 600 mm, and finally the resistance during the relaxation and recovery of the CFRP sample was measured for 10 s to allow for the recorded resistance readings to stabilise.

## 3. Results and Discussion

### 3.1. Conductivity Optimisation

The CFRP layer was initially found to be conductive, hence the insulator layer was printed and UV cured on the CFRP sample. The performance of the insulator layer was evaluated, and upon curing, the surface resistivity of the ink measured 8.2 × 10^12^ Ω/sq, which is adequate for the insulative purposes of this research [32,33]. Additionally, a simple two-point-probe test where one probe was placed on the cured insulator, and the other was placed on the surrounding CFRP was done. As the probe did not measure a conductivity, the insulator layer was determined to successfully prevent interconnects between the silver ink and the CFRP.

**Figure 4:**
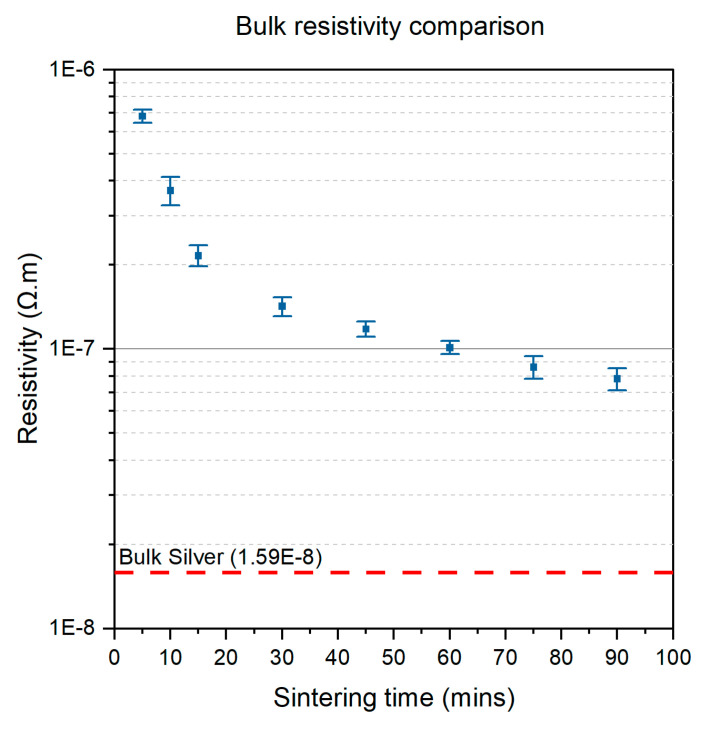
Conductivity improvement of the silver NPs when sintered at 160 °C up to 90 min.

The conductivity optimisation of silver using the 15 × 15 mm^2^ test samples is shown in Figure 4 below. Using the four-point probe, the sheet resistance of the samples was obtained, after which the sheet resistivity, $\rho_{s}$, can then simply be calculated using by multiplying the sheet resistance by the thickness, t, of the printed pattern as shown in Equation (1), and the sheet resistivity value can be compared to the bulk resistivity value of silver ($1.59\times 10^{-8}\;\Omega \cdot m$).

(1) $$\;\rho_{s}=R_{s}\times t$$

The optimum sintering duration for the silver NPs printed on the CFRP laminate was chosen to be 90 min. The pattern changed from a matt grey to shiny silver once the organic dispersant was decomposed. At the start of the sintering, there is an exponential decrease from 30 min to 60 min, after which the curve plateaus, indicating that the decrease in resistivity is less prominent, particularly after 60 min. The change in resistivity measured at *t*_sint_ = 5 min → *t*_sint_ = 75 min indicated a 87.38% decrease, whilst the change in resistivity measured from *t*_sint_ = 60 min → *t*_sint_ = 75 min was only 2.21%, and *t*_sint_ = 75 min → *t*_sint_ = 90 min was only 1.15 %. As the change in resistivity was minimal, the optimized duration was chosen within 75–90 min for the performance requirement of the strain gauge on the CFRP [34,35,36]. The improvement in conductivity is significant in the first 15 min while the additional 75 min allowed an increase in conductivity by almost an order of magnitude. In some cases, an overall sintering time of 60 min was sufficient to obtain a conductive pattern. The maximum bulk conductivity value obtained as compared to bulk silver is 22.1%.

Further SEM analysis of the surface of the sintered silver NPs indicated significant neck formation between the NPs, which explained the improvement in conductivity. In the first stage of sintering, the application of thermal energy is used to first evaporate the solvent. Then, the nanoparticle stabilizers are decomposed, which indicates the onset of sintering. The nanoparticles are free to move, thereby forming a percolating network of NPs. It is at this stage that necks are formed in the printed pattern. The additional thermal energy input in the system is then used for the growth of necks, while simultaneously improving the sintering process through particle migration.

**Figure 5:**
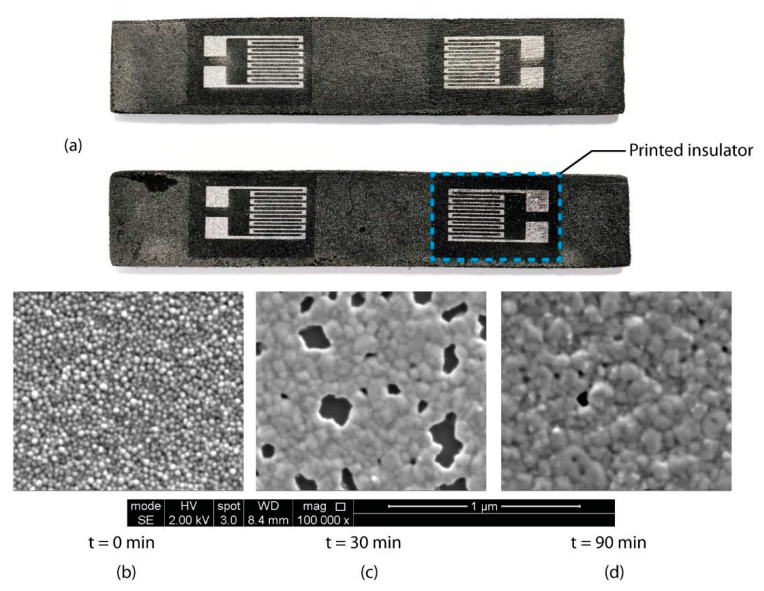
(**a**) Snapshot of the strain gauges printed on the CFRP sample. Results of SEM analysis at different stages of sintering of the silver nanoparticle ink; (**b**) represents the printed silver NPs ink without any heat treatment; (**c**) represents the surface of the pattern after 30 min of sintering, indicating necking and a porous surface; (**d**) represents the final sintered pattern after 90 min, showing a significant reduction in porosity.

The thickness of the printed strain sensor measured 195 nm for two layers of silver ink printed onto the insulator at a droplet spacing of 80 µm. As the sintering process began, the constituents of the silver ink aside from the nanoparticles (such as the solvent and the dispersant) were removed from the printed film through the application of heat. As the solvent in this case is triethylene glycol monomethyl ether which has a boiling point of 122 °C [37], the sintering temperature of 160 °C was sufficient to initiate evaporation and the breakdown of the dispersant ligaments, thereby freeing the silver nanoparticles. At this stage, the Ostwald ripening process can begin [38], and necking within the nanoparticles initiates. However, at this stage, the porosity of the printed silver film is rather high (11.1 %) as shown in Figure 5c due to the incomplete sintering process. The porosity of the printed film decreases as the sintering process is extended to 90 min, which allows time for the lattice diffusion of the nanoparticles within the grain boundary to form a dense percolating network, as explained by Greer et al. [39]. At the end of the lattice diffusion process, the porosity of the printed film is the minimum at 2.80%, yet a perfectly non-porous film is rather difficult to achieve at ambient conditions due to the presence of contaminants such as dust particles. Additionally, due to the grain boundary relocation, the printed film experiences some shrinkage, albeit minimal at only 3.37% based on a digital comparison on the samples after sintering with the actual dimensions of the strain sensor in Appendix E, particularly in the planar *x-y* orientation due to the relocation of the nanoparticles within the lattice structure [40].

Figure 5a represents a snapshot of the strain gauges printed on the CFRP sample, and Figure 5b–d are SEM micrographs of the printed silver NP ink, which indicate an increase in particle diameter due to the agglomeration of the nanoparticles. In Figure 5b, the sample had not been subjected to any heat treatment, indicating the actual shape and size of the nanoparticles present in the ink (≈50 nm). Figure 5c was subjected to a heat treatment of 160 °C for 30 min which shows the increase in particle size. It also shows the neck formation at distinct locations within the sample, which incidentally indicates a porous surface. Figure 5d was thermally sintered at the same temperature but for a total of 90 min. At this stage, the porosity of the sample can be observed to have decreased significantly from an average of 11.1% to 2.80% from 30 min–90 min, indicating a 97.2% dense structure based on an average of five samples. The size of the nanoparticles increased, resulting in an average of 162.90 nm in diameter, and a distribution range between 117–297 nm. 

### 3.2. Adhesion Classification

**Figure 6:**
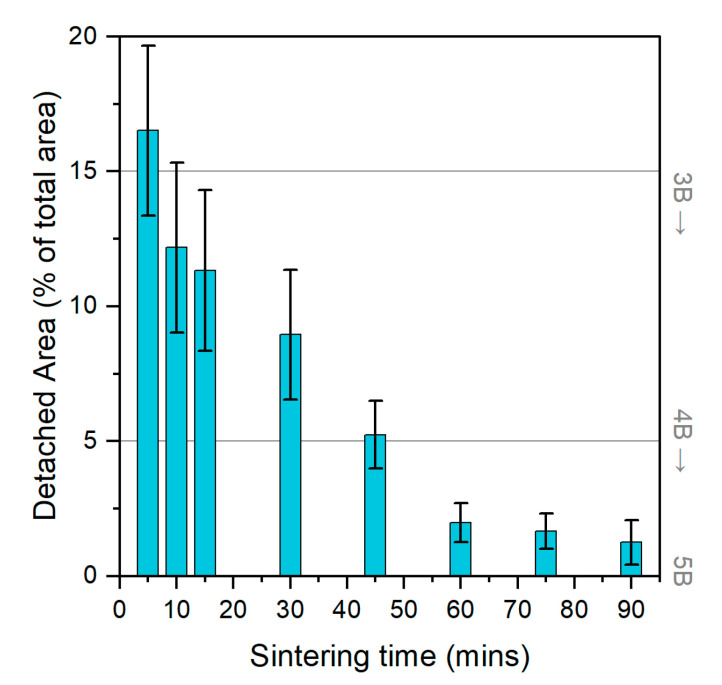
Representation of the adhesion evaluation in terms of the detached area as a percentage of the total printed area (15 × 15 mm^2^) for the silver nanoparticle-based ink. The right y-axis represents the adhesion classification based on the F1842-15 standard, starting from 5B to 4B and 3B in this set of experiments.

An adhesion test is often overlooked in the field of printed electronics, but it is crucial to evaluate this property for understanding the interface between the printed pattern and the substrate, especially for multi-layered patterns. This additional step of evaluating the adhesion is crucial for understanding the lifespan of the printed feature, particularly when it will be exposed to external harsh environments, which may cause defects or delamination due to excessive deformation. The adhesion of the 15 × 15 mm^2^ test samples is shown in Figure 6 below. As the duration of the sintering was increased, the adhesion of the samples improved from a classification of 3B to 4B, with the most conductive samples achieving an average delamination of only 1.25% compared to the overall printed area on the insulator ink. After 5 min of sintering, the adhesion of the printed pattern was classified at 3B due to the removal of between 13–18% of the silver nanoparticle layer. As the sintering time was increased, the adhesion was seen to improve from 3B to 4B within 45 min of sintering at 160 °C, while simultaneously resulting in an increase in conductivity due to the formation of necks within the nanoparticle layer, as shown in the previous SEM image in Figure 5b. Finally, as the samples were left to sinter for a total of 90 min, the delaminated area decreased from an average of 5.24% to 1.63%, hence they were classified as 4B. 

There are several factors that affect the adhesion at the interface of the printed film and substrate, such as surface roughness, surface energy and porosity. Larsson et al. [41] described the main adhesion mechanisms, such as chemical bonding, mechanical interlocking and electrostatic. The mechanical interlocking phenomenon occurs when the metallic nanoparticles are anchored within the microscopic cavities of the insulator surface, as explained by Larsson et al. [41]. In this case where a metallic nanoparticle-based ink was printed on a non-porous surface, mechanical interlocking is the most dominant adhesion mechanism as there was no surface treatment done prior to printing. This is supported by Halonen et al. [42], Niittynen et al. [43] and Gopal Kirtania et al. [44] who also reported that the sintering time and sintering duration are the most influential factors for adhesion improvement.

Additionally, the use of image processing software to calculate the detached area eliminates the subjective bias and inaccuracies due to human error. In the research output by Lukacs et al. [30], they compared results obtained from different individuals and found that the classification of the adhesion results in the mid-range (between 3B and 4B) was subjective and varied across different individuals. As a solution, they introduced an image processing software to evaluate the sample, which eventually eliminated the human bias and provided a more rigorous, systematic evaluation procedure.

The most common disadvantage of film sensors which was highlighted by Rocha et al. [27] is the poor adhesive bonding of the sensors to the host structure. In the case of film sensors that are attached to the CRFP, by means of an adhesive layer (e.g., epoxy-based adhesive, polyester or cyanoacrylate), the delamination of the sensor from the CRFP is the main cause of signal failure. As an illustration, Komurlu et al. [45] found that up to 51% of strain loss can be observed if the choice of the adhesive type is not taken into account.

Embedded sensors mitigate this delamination problem since they are directly embedded into the CRFP. However, some important considerations must be made since the CRFP layer is conductive. In our study, the use of an insulator layer prevented the formation of shorts and interconnects whilst delivering optimum adhesion of the printed sensor to the insulator layer. Additionally, the use of embedded sensors eliminates the requirement for an interfacial adhesive and also a substrate, hence minimizing two layers, which can measure up to 200 µm in thickness. In an application context, this decrease in film thickness can contribute to an overall sensor geometry reduction of up to 90% per sensor array.

The advantages of the embedded sensors extend to an improved adhesion classification, as shown in Figure 6 above. In this case, the 4B adhesion classification of the sensors to the CRFP indicates superior bonding. Subrahmanya et al. [46] varied the thickness of the adhesive layer for strain gauges to investigate the effects on the reliability of the strain measurements. The authors reported a decrease in signal reliability with an increase in adhesive layer thickness. Hence, based on these findings, the embedded sensors in the CFRP improve the sensor signal fidelity by ensuring both a superior bond as well as a thin geometry, which was achieved by the optimized inkjet printing process.

### 3.3. Sensor Performance after Induced Strain

In order to verify the efficacy of the printed strain gauge, the samples were tested on a bending rig as noted in the methodology section. Following the methodology, the results were collected and analysed following two Equations (2) and (3):

(2) $$\Delta R_{Abs.}=R_{600mm\;}\left(pre-strain\right)-R_{n}\left(post-strain\right)$$

(3) $$\Delta R_{Rel.}=R_{n}\left(pre-strain\right)-R_{n}\left(post-strain\right)$$

where *n* represents the sequence of the test. In this experiment, the tests were performed from a low strain region and proceeded with increasing strains (i.e., r = 600→ 500→ 400→ 300 mm radius bending rigs).

**Figure 7:**
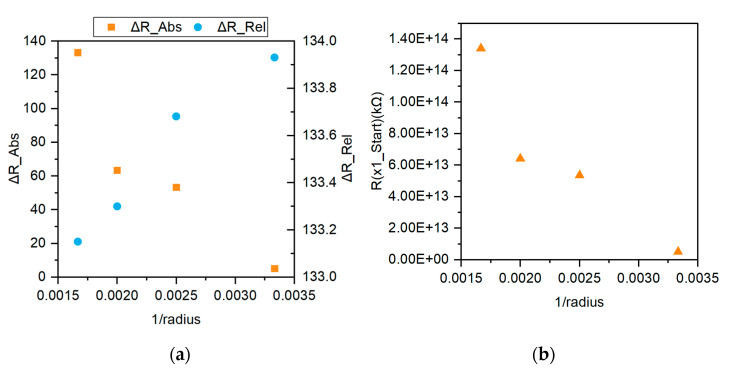
(**a**) Plots of the change in resistance using two different resistance references: Pre-strain (for the virgin sample) and pre-bending (for each test sequence); (**b**) Pre-bending resistance for the inverse bending radii of 600, 500, 400, and 300 mm.

The results for both schemes are shown in Figure 7a and are plotted against 1/*r* where it is observed that $\Delta R_{Abs.}$ increases as strain (equivalent to 1/*r*) increases. The reverse is true for the relationship of $\Delta R_{Rel.}\;$against 1/*r* where $\Delta R_{Rel.}\;$decreases with increasing strain. This may be attributed to the hysteresis behavior exhibited by the sensor. Figure 7b shows values of $R_{n}\left(pre-strain\right)\;$for the samples plotted out against 1/*r* where it is observed that a decreasing resistance is being recorded for each subsequent test. This reduction in initial resistance suggests that the strain gauge is experiencing increasing compressive stresses in its pre-strain stage. This may be due to enlarged interparticle straining arising from the cyclical strain [47]. Furthermore, in the case of CFRP which are fabricated using thermoplastics, the presence of the fibers within the matrix will cause a decrease in the elongation as the fibers hold the structure firmly in place [48]. 

Rocha et al. [27] summarized the specifications for SHM sensors in five criteria, namely:

1. The sensors should monitor the real-time structural performance of the structure and be immune to external factors;
2. The signal transmission from the sensor to the data acquisition device should be reliable;
3. The embedment of the sensor should not be detrimental to the performance of the structure;
4. The lifespan of the sensors should be parallel to the lifespan of the structure;
5. The sensors should be easily commissioned on the structure, including minimizing the risk of delamination.

In comparison with the criteria by Rocha et al. [27], the integration of the printed sensor within the CFRP was successfully done with inkjet printing technology, and the sintering optimization ensured a high-performance sensor in a time-efficient manner. Moreover, the fragility aspect of integrating the sensors was circumvented by using functional inkjet inks, also ensuring the light-weighting and reduced dimensionality of the overall structure. The sensor was able to detect the change in strain during loading and unloading, thereby highlighting the real time monitoring performance together with the CRFP. Further work can be aimed at improving the quality of the signal transmission by incorporating a printed wheatstone bridge circuit within the CFRP [49]. For adequate implementation in SHM in an industrial context, additional work can be done to detect the damage location, damage type, and size of damage [50,51]. In terms of adhesion classification, an encapsulating layer comprising a silicone elastomer can be added to protect the exposed surface from external factors such as high stress dynamic environment and friction. Eventually the additional encapsulation layer will improve the lifespan of the sensor [52,53]. 

## 4. Conclusions

In this study, an all inkjet-printed sensor was successfully embedded directly on a carbon fiber reinforced polymer (CFRP). In order to obtain a good sensor, which detects the actuation (in this case, a strain gauge detecting the applied strain), the inkjet printing parameters were optimized to yield a conductive pattern without resulting in any delamination during testing. The inkjet printing parameters were optimized to yield a printed sensor with comparable conductivity, adhesion and performance during actuation. The optimization process involves a series of sintering tests, resulting in a maximum bulk conductivity of 22.1% as compared to bulk silver when the sintering was performed at 160 °C for 90 min. Further SEM imaging showed a reduction in voids from 11.1% to 2.80% after optimum sintering. The adhesion of the samples is also critical to the long-term performance of the sensor, which was quantified based on the ASTM F1842-15 standard for printed electronics through the cross-cut test. The embedded sensors achieved an adhesion classification of 4B, which ensures superior bonding as compared to using adhesives for attached sensors. As stated by Rocha et al. [27], the delamination of attached sensors to the host structure is the main source of failure, which was circumvented in this study. Finally, a sensor in the form of a strain gauge was printed directly on the CFRP sample with the aim of improving the fidelity of the sensor and also avoiding sensor degradation due to the addition of mid-interfacial adhesive layers. The sensor performance was analyzed using a bending rig which applied a pre-determined level of strain, and the change in resistance was measured using a digital meter. The results indicate a good response of the sensor to the applied strain, as the absolute resistance increases as the strain increases, and vice-versa. In conclusion, this study shows that inkjet printing can successfully be used as a technique to embed a sensor directly in a CRFP sample, with the aim of targeting structural health monitoring through non-destructive testing in real-time operation.

## Figures and Tables

**Figure A1:**
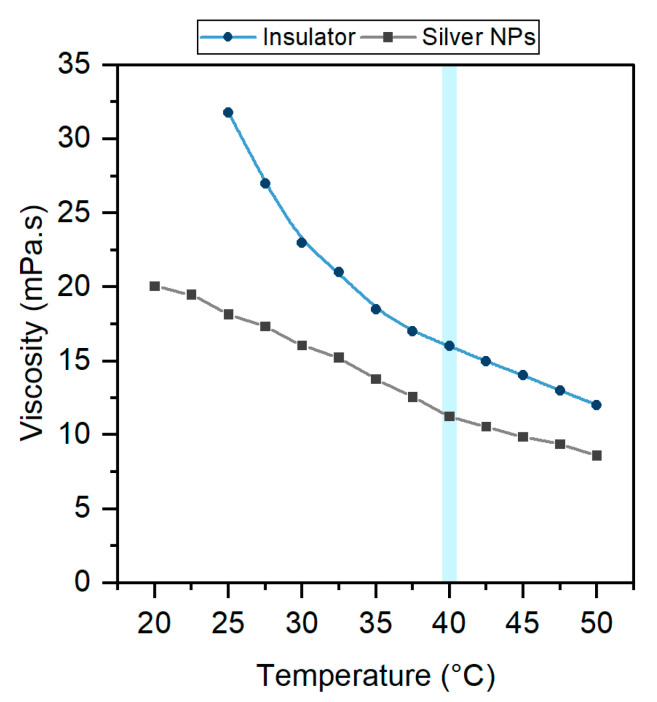
Plot of the viscosity variation of both the functional silver nanoparticle and the insulator inks as a function of temperature. The printable region chosen was at 40 °C, highlighted in light blue on the plot.

**Figure A2:**
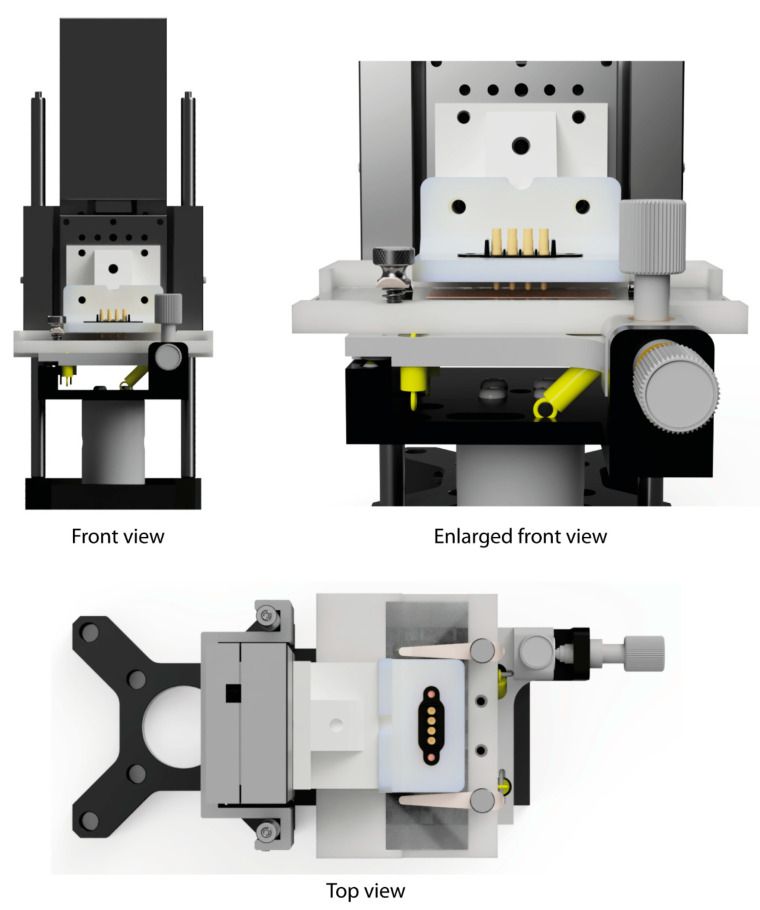
Four-point probe measurement station.

**Figure A3:**
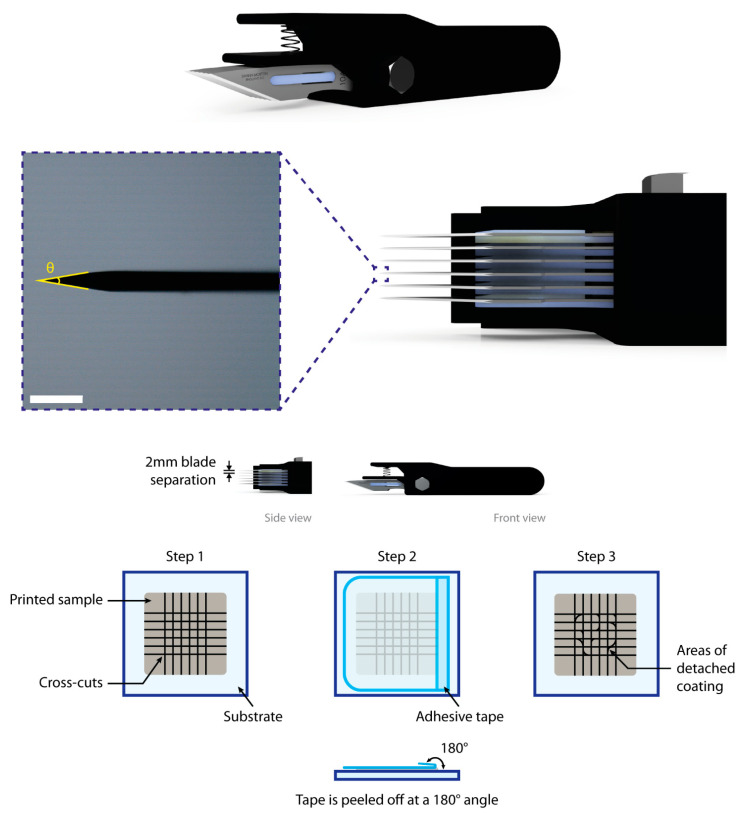
Illustration of the cross-cut procedure, showing an in-house fabricated cutter used to maintain a 2 mm separation between the blades, and the 3-step process of evaluating the adhesion of the printed sample.

**Figure A4:**
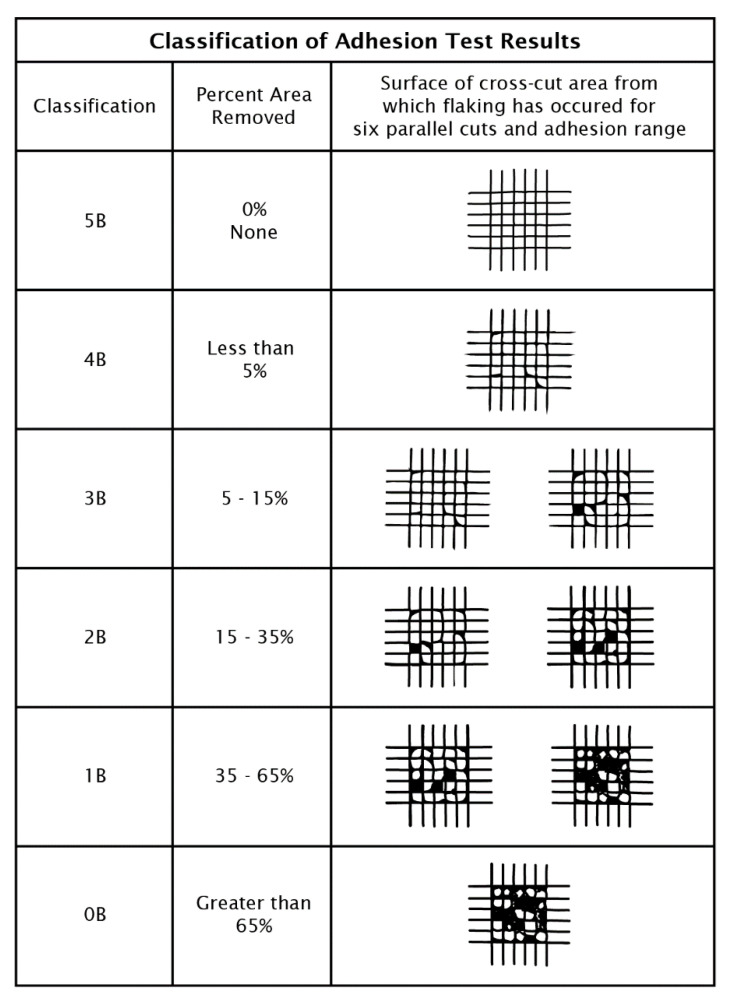
Classification of the different adhesion levels as a result of the cross-cut test.

**Figure A5:**
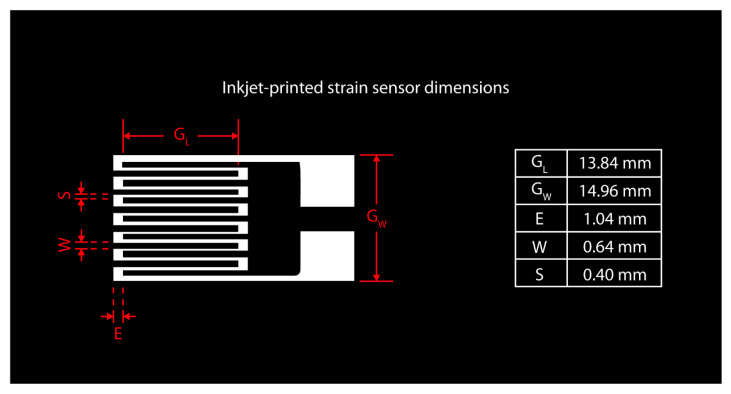
Strain sensor dimensions.

## Data Availability

The data presented in this study are available on request from the corresponding author P.J.S.

## Appendix A

**Table A1:** Details of relevant ink properties used throughout the research.

Ink	Viscosity (cP)	Particle Content wt. %	Recommended Sintering Temp.	Recommended Sintering Time	Manufacturer
INI	16.00 at 40 °C	-	- * UV	- * UV	Dycotec Materials (DM-INI-7003)
AgNPs	11.25 at 40 °C	30–35	150 °C	30 min	Sigma-Aldrich (no. 736465)

* The insulator ink can only be cured by UV at the specified emission wavelength.

## Appendix D

ASTM F1842-15 Standard Test results classification.
